# Identification and Spatial Analysis of Co-Occurring Pollution Sources of HCHs, PCBs, and PFASs in the EU and Potential Risks of Soil Pollution

**DOI:** 10.1021/acs.est.6c01518

**Published:** 2026-05-23

**Authors:** Naila Hina, Juliane Glüge, Martin Scheringer

**Affiliations:** Institute of Biogeochemistry and Pollutant Dynamics, 27219 ETH Zürich, 8092 Zürich, Switzerland

**Keywords:** soil pollution, polluting activities, organic pollutants, PFASs, cocontamination

## Abstract

Soil contamination represents a major environmental concern for maintaining soil health. Among various contaminants, persistent organic pollutants (POPs) are particularly problematic due to their persistence, bioaccumulation potential, and toxicity. Anthropogenic activities significantly contribute to soil contamination with various chemicals. This study provides an overview of activities contributing to the contamination of soil by hexachlorocyclohexane isomers (HCHs), polychlorinated biphenyls (PCBs), and per- and polyfluoroalkyl substances (PFASs). EU-wide databases were used to map spatial patterns, co-occurrence of multiple pollution-causing activities at a high resolution of 1 km^2^, and potential cocontaminated areas. There are approximately 385 locations where sources of PCBs, HCHs, and PFASs co-occur within 1 km^2^, with 10% of these locations identified as potentially cocontaminated with more than one pollutant. The identified activities can serve as a starting point for EU Member States that are still in the process of creating, or planning to create, an inventory of historical and current pollution-related activities associated with soil contamination. Furthermore, the results serve as proof of concept to demonstrate the spatial co-occurrence of pollution-causing activities posing risks of cocontamination. The evidence provided by these data sets supports soil monitoring, targeted decision making, and environmental management practices.

## Graphical abstract

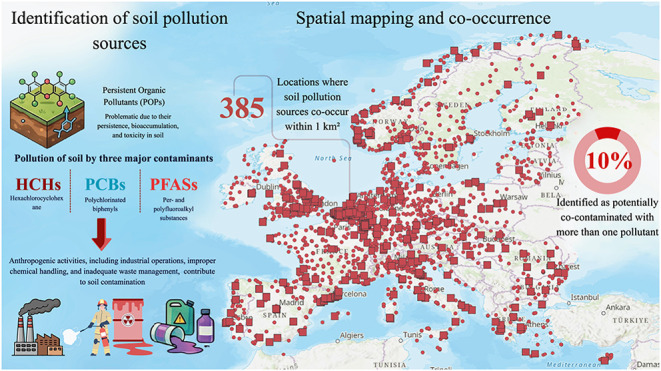

## 1 Introduction

Persistent organic pollutants (POPs) are commonly referred to as “poisons without passports”.^1^ They are a group of organic chemicals that are hazardous due to their resistance to environmental degradation, transboundary movement, significant harmful effects on human and environmental health, and bioaccumulation, which might result in biomagnification-induced death in top-ranked biota.^1^ International restrictions, such as those imposed by the Stockholm Convention, aim to limit the production and use of POPs. However, despite these regulations, many soils remain contaminated owing to historical usage and ongoing production of POPs as byproducts of industrial processes, waste disposal, and agricultural activities.^2^ Although restrictions have been implemented, they have not been effective in mitigating soil contamination in the long term.

Hexachlorocyclohexanes (HCHs), polychlorinated biphenyls (PCBs), and per- and polyfluoroalkyl substances (PFASs) are all relevant as toxic and persistent chemicals under international frameworks. γ-HCH and PCBs were among the initial 12 POPs in the Stockholm Convention, reflecting their common properties of persistence, bioaccumulation, and adverse effects on human and environmental health.^3^ Many PFASs also pose adverse effects on human health, and many exhibit mobility from soil and cause groundwater contamination long after initial soil deposition. Similarly, HCH production at numerous sites produced millions of tonnes of waste and unintentional byproducts of lindane manufacture (mostly α- and β-HCH) dumped on landfills. Further, HCH spreads beyond its point of origin during landfill operations through volatilization and dust dispersal to adjacent soils.^4–6^ This means that, even with reduced current usage, legacy contamination of these pollutants remains a concern.

PCBs are a class of POPs and were among the initial pollutants listed by the Stockholm Convention. Due to their widespread use in industrial applications such as electrical equipment, paints, plastics, rubber, PVC coatings, and hydraulic systems, PCBs have become pervasive in various environmental matrices, including soil. They are commonly found in high concentrations (range from 260 ng g^–1^ to 6.722 × 10^6^ ng g^–1^) in industrial zones,^7^ primarily due to the use of their commercial products. Additionally, informal electronic waste recycling,^8^ historical accidental events, such as damage to capacitors, and waste from electrical equipment such as transformers^9^ remain major sources of soil pollution. Once PCBs are present in the soil, their mobility is influenced by organic matter content, soil properties, and tillage practices, with higher chlorinated congeners persisting longer in soils rich in organic matter.^10,11^ Therefore, contaminated soils act as reservoirs of PCBs and facilitate their transfer to plants and food chains.

HCHs are a mix of several isomers with the γ-isomer, lindane, acting as an insecticide, and they form another group of pollutants causing widespread soil contamination. The primary sources of HCHs are industrial activities, including HCH production, processing, accidental spills or leakage from storage facilities, improper waste management, and agricultural use.^6,12,13^ A huge amount of HCH waste has been produced and dumped across sites in the EU, resulting in soil contamination at these locations, such as 4000 tonnes of HCH waste in the Netherlands that contaminated 200,000 tonnes of soil, and 82,000 tonnes of dumped HCH isomers that caused between 500,000 and 1 million tonnes of contaminated soil in Spain.^14^

PFASs are a group of persistent synthetic chemicals that have also become a significant environmental concern due to their extreme persistence and health risks. PFASs are used in many different applications and in many consumer products. In total, over 200 use categories and subcategories have been identified for more than 1400 individual PFASs.^15^ Due to their diverse uses, the sources of PFAS soil pollution are also diverse. The most elevated levels of PFASs in soil were found at sites exposed to aqueous film-forming foam (AFFF), PFAS production facilities, and biosolids.^16^ The highest concentrations of PFASs in soil, ranging from 211 ng g^–1^ to 3.57 × 10^5^ ng g^–1^ dry weight, were found in samples collected from fire training locations.^17–19^ PFAS contamination in soil poses a persistent challenge for environmental quality and public health. PFAS contamination in soil can cause exposure of humans to PFASs via contaminated drinking water or by eating contaminated meat or fish. This exposure is linked to several health-related issues such as breast cancer, infertility, increased cholesterol, diabetes, cardiovascular diseases, altered metabolism, thyroid issues, vitamin D deficiency, atherosclerosis, and osteoporosis.^20,21^

The term "soil contamination" refers to the presence of harmful substances in soil above background levels, which may reduce the quality and functionality of soil and typically triggers further investigation. In contrast, "soil pollution" is the presence of toxic substances in soil at levels that cause adverse effects on soil productivity, ecosystems, and health.^22,23^ The background contamination values for PFASs, HCHs, and PCBs vary and are often influenced by industrial activities, atmospheric deposition, and long-range transport; e.g., the background values of PFASs globally in soil range from 0.25 to 19.5 ng g^–1^ with higher levels near industrial areas.^24–27^ Some guideline values exist for PCBs (0.08 to 1.0 mg/kg for total PCBs) and HCHs (< 50 ng/g),^28–30^ but there is significant variability across regions, and global standards for PFASs in soils are lacking. All of these pollutants have bioaccumulation potential and toxicity to soil organisms. PFASs significantly affect soil microbial communities and other soil organisms such as earthworms.^31^ PFASs and PCBs are absorbed by plants from contaminated soil and are ingested by animals, thereby facilitating biomagnification across food webs.^32,33^ The inefficient process of HCH production generates about 10 tonnes of HCH waste isomers with every one tonne of lindane produced. The inadequate management of the resulting waste can lead to widespread contamination of soil by HCH and to toxic effects of HCH isomers on the soil microbiome.^34,35^

Several pollution sources for HCHs, PCBs, and PFASs exist in the EU. Understanding the sources of pollution, the complex interactions between these sources, their distribution, and the suitabiliy of remediation strategies is important for managing soil contamination. A number of studies have focused on individual groups or substances of pollutants and their pollution sources.^36,37^ Several activities, including industrial operations, improper chemical handling, and inadequate waste management, significantly contribute to soil contamination. Understanding the specific activities causing soil contamination is critical for effective mitigation strategies. Furthermore, it is also important to identify the spatial distribution and overlap of these sources to determine where co-occurrence might intensify soil pollution. The aim of this study is to identify historical and current pollution-related activities associated with soil contamination by HCHs, PCBs, and PFASs. The spatial distribution of these activities is mapped to see the extent of the contamination and to identify areas where more than one of these activities occurs within grid cells of 1 km^2^ size. The identification of activities as potential sources of soil contamination will support the efforts that are included in the EU’s Directive on Soil Monitoring and Resilience (soil monitoring law), which states that “Member States shall systematically and actively identify all sites where a soil contamination is suspected based on evidence collected through all available means (“potentially contaminated sites”)”.^38^ The approach of spatial distribution mapping will assess the likelihood of multi-pollutant soil contamination and help to prioritize monitoring and remediation efforts. This study focuses exclusively on soil contamination sources and does not include pollutant concentration data; therefore, no differentiation between soil contamination and soil pollution, which is solely based on concentrations, is made. The contribution of this approach is that it can guide a systematic way to identify potentially contaminated sites, nontargeted and extensive soil analyses, especially in areas with multiple pollution activities associated with more than one pollutant. Such analyses can detect both known and suspected or unknown contaminants, thereby supporting the EU’s commitment to environmental protection.

## 2 Methods

### 2.1 Identification of Pollution Sources

The activities causing soil pollution by HCHs, PCBs, and PFASs in the EU were identified according to the following approach: Selected were only activities with a high likelihood of causing soil pollution of the selected substances. Initially, a systematic literature search was conducted and combined with expert knowledge to search for activities. Subsequently, activities were added that have been identified in national programs as activities causing soil contamination. Finally, the list obtained in this study was compared against the lists of activities that have been used in the national programs of the different states to identify potentially contaminated sites.

### 2.1.1 Systematic Literature Search

The literature search was conducted using the Web of Science (WOS) to identify studies reporting polluting activities associated with soil contamination. The following keywords were used:

“polluting activities” AND “soil”,

“polluting activities” AND “soil” AND “polychlorinated biphenyls”,

“polluting activities” AND “soil” AND “per- and polyfluoroalkyl” AND “PFASs”, and

“polluting activities” AND “soil” AND “hexachlorocyclohexanes” AND “HCHs”.

A search was also carried out in Google with two steps. First, it was investigated which activities can potentially lead to soil contamination for a certain substance or substance group. This was done by searching with the keyword “source” and the substance class name or individual substance names. In the second step, literature was searched that confirmed that those activities have really led to soil contamination. This was done by searching for the activity and substance name combined with the word “soil”. Google was also used to find hits from reports and other gray literature, and not only from scientific publications. Additionally, for PFASs and PCBs, we went back to our own research from the past^15,39^ and added activities.

### 2.1.2 National Programs of Potentially Contaminated Sites

Many of the 27 EU Member States, the 4 European Free Trade Association (EFTA) Member States (Iceland, Liechtenstein, Norway, and Switzerland), and the United Kingdom (UK) have national programs to identify potentially contaminated sites. For each country, we tried to identify whether the country has already identified potentially contaminated sites and published relevant activities, and if the activities are substance-specific and might therefore be relevant to our work. The activities identified for the different countries were then compared to see whether there are common activities that are used by all countries and should also be added.

### 2.1.3 National Programs of Remediated Sites

Data on the remediation of contaminated hotspots in the EU were collected for an accompanying study.^40^ We searched for soil remediation data in 30 European countries and contacted environmental agencies in 18 of them. Details on the data collection and availability are provided in the previous study.^40^ The collected data were used to identify any new activities that had not previously been reported as sources of soil pollution in the literature. An activity was added only if it was reported to cause pollution from the specific pollutant of focus in this study.

### 2.2 Data Collection for Mapping the Identified Activities

After identification of activities causing HCH, PCB, and PFAS soil pollution, the next step was to collect data on where these activities are present in the EU, to map the spatial extent of these activities. Data were collected for mapping the activities identified from various sources, including national and international inventories, and the European Pollutant Release and Transfer Register (E-PRTR).

### 2.2.1 European Pollutant Release and Transfer Register (E-PRTR)

E-PRTR is a Europe-wide register containing information on the annual release of pollutants from industrial facilities into air, water, and soil, as well as the transfer of pollutants present in wastewater and waste. It is a public database covering information on the EU Member States, Iceland, Liechtenstein, Norway, Serbia, Switzerland, and the United Kingdom as of February 2020. The database covers 91 pollutants listed in Annex II of Regulation (EC) No 166/2006,^41^ including greenhouse gases, other gases, heavy metals, pesticides, chlorinated organic substances, and various inorganic substances. However, emission data for many of the PFASs that have been found in the environment, e.g., perfluorooctanoic acid (PFOA) or perfluorooctanesulfonic acid (PFOS), are not available in the E-PRTR. According to the regulation, facilities engaging in any of the 65 activities listed in Annex I of Regulation (EC) No. 166/2006 are required to report releases when these exceed specified thresholds. The activities listed in Annex I cover mainly energy production, production and processing of metals, mineral industry, chemical industry, waste and wastewater management, paper and wood production and processing, intensive livestock production and aquaculture, animal and vegetable products from the food and beverage sector, and other activities such as producing textiles and leather tanning.

Here, data were retrieved from the E-PRTR for activities reporting emissions of PCBs and/or HCHs into any environmental matrix (water, air, and soil). Duplicate entries reporting the emissions of the same pollutant in multiple matrices were excluded. Additionally, data were retrieved for facilities that, despite not reporting emissions of PCBs and/or HCHs, have been identified from the literature as potential sources of soil contamination by these pollutants.

### 2.2.2 National and International Inventories

The “Forever Pollution Project” is an international and interdisciplinary journalism investigation to track PFAS pollution across Europe.^37^ The data set collected during the project is now hosted by the National Centre for Scientific Research (CNRS) as the PFAS data hub. The PFAS data hub contains data on four categories, including PFAS producers, known sites of PFAS contamination, known PFAS users, and presumptive contamination sites. Data from CNRS on PFAS producers were retrieved and included in this study. No data were included from the known sites of PFAS contamination and PFAS users because it was not clear which activities were causing the pollution, nor whether the contamination was in soil or groundwater. From the presumptive contamination sites, only those sites were included where the pollution-causing activity has been identified in this work as sources of PFAS contamination in soil.

Another EU-wide data inventory, the “Inventory of sites potentially impacted by hexachlorocyclohexane (HCH)”,^36^ was used to extract the data on HCHs. The project assessed the presence of HCHs in the EU and developed an EU-wide inventory of sites where HCHs and lindane were handled. The country-specific reports were retrieved to extract the data on the type of activity and the coordinates of the locations of these activities.

Data obtained from national inventories online or upon request were also included in this study. The data received from most of the countries included potentially contaminated sites without information about the pollutant and pollution-causing activities. Therefore, only data from countries that provide information on pollution activities and relevant pollutants, or that lack pollutant information but have one of the activities we identified here as sources of soil pollution and that include spatial information, are included here.

### 2.3 Data Processing and Spatial Analysis

For mapping the pollution sources of PFASs, PCBs, and HCHs, the collected data were prepared for an exploratory data analysis (EDA). Data from all sources mentioned above were compiled and cleaned for missing and duplicate entries. The following parameters were extracted from all data sources to ensure harmonization:

1. Pollution source locations as latitude and longitude, standardized to the coordinate system “World Geodetic System 1984” (WGS84). Pollution source locations lacking coordinate data were excluded from the database. For data provided as shapefiles, coordinates were derived using the geometric calculations in ArcGIS Pro 3.4. Additionally, centroid coordinates were calculated to maintain a single entry for the sites represented as polygons.
2. Polluting substance/substance group associated with each location.
3. Additional parameters, such as country information and data sources, were also used.

For the spatial analysis, ArcGIS Pro 3.4 was used. The initial mapping of pollution sources was conducted to delineate the spatial extent of the data. The coordinates were reprojected using the projection tool of ArcGIS to transform the coordinate system from WGS84 to UTM (Universal Transverse Mercator). The WGS84 coordinate system represents spatial positions using latitude and longitude in degrees, whereas UTM represents the Earth’s surface in a planar format and uses meters as its unit of measurement. This transformation was necessary for dividing the study area into equal cells to facilitate accurate spatial analysis and mapping. In the next step, the area was divided into 1 × 1 km^2^ cells utilizing the “create fishnet” tool in ArcGIS Pro 3.4. Subsequently, the “spatial join” and “summarize within” tools were employed to calculate the number of activities within each cell (1 × 1 km^2^) and to map the squares where activities co-occur that contribute to soil pollution with either single or multiple pollutants.

### 3 Results and Discussion

#### 3.1 Identified Pollution-Causing Activities

Table 1 lists the activities causing PFAS, PCB, and HCH pollution that have been identified through the literature search and expert knowledge. They include four main activities for PFASs, nine for PCBs, and six for HCHs. No new activity that clearly causes soil pollution was identified from national programs of contaminated sites other than those activities already covered by the literature search and expert knowledge.

**Table 1:** List of Activities Identified in the Literature as Sources of Soil Pollution for Per- and Polyfluoroalkyl Substances (PFASs), Polychlorinated Biphenyls (PCBs), and Hexachlorocyclohexanes (HCHs)

**pollutant**	**type of pollution activity**	**refs**
**PFASs**	production of PFASs, including fluoropolymers	^19,42,43^
	use of fire-fighting foam at airports, military sites, and firefighting training sites	^19,44,45^
	deposition/application of compost or sewage sludge on fields	^46,47^
	landfilling and waste handling	^46^
**PCBs**	former PCB production sites	^48–50^
	recycling of ferrous scrap	^51^
	shredding of electronic waste	^51^
	storage site for discarded electrical equipment, including transformers	^52,53^
	renovation of anticorrosion coatings without emission-reducing measures	^54^
	petrochemical industry	^55^
	manufacture of paints, lacquers, and varnishes	
	thermal power stations and other combustion installations	^56^
	landfilling and waste handling	^50^
**HCHs**	HCH production sites (sites where technical HCH and/or lindane were produced)	^13^
	processing sites (sites where technical HCH and/or lindane were processed and formulated into market-ready pesticides)	
	storage facilities (sites with storage facilities where obsolete stocks of POP pesticides, including lindane and HCH and its degradation products, are stored awaiting final disposal)	
	treatment centers (sites where HCH and its degradation products are/were treated and/or destroyed in the scope of projects mitigating the related environmental risks)	
	wood treatment sites	
	landfilling and waste handling	

The results from national programs that have identified potentially contaminated sites, for 19 out of the 32 EU and EFTA countries + UK, are shown in Table S1 in the Supporting Information (SI). Substance-specific polluting activities were investigated and defined in detail only in a few countries, including Belgium (Flanders), Cyprus, France, Norway, Poland, Sweden, and Switzerland. However, each country has its own list of activities, and the specified activities vary a lot. The number of included activities and the level of detail with which these activities are described vary considerably among the countries. Therefore, no activities were added on the basis of their occurrence in one of the national lists alone. We used the activities from the national programs mainly to confirm the polluting activities that were gathered through the literature search and expert knowledge.

Table 1 provides an initial overview of activities contributing to hotspot pollution in several countries in the EU and EFTA. While some important activities may have been missed because of the limited scope of outcomes from the sources used, the compiled list serves as a valuable starting point for developing national inventories of polluting activities. Importantly, the activities included were confirmed through literature sources, providing a reliable foundation for identifying potentially contaminated sites. Therefore, member states should consider using the list of potentially contaminating activities identified for PFASs, PCBs, and HCHs when compiling national inventories of contaminated sites and conducting preliminary site screenings. It is important to note, however, that this list is for soils only. There are more activities that can lead to ground and surface water contamination. These activities (e.g., chrome plating or paper and textile production for PFASs) are not included here.

#### 3.2 Data Summary of Sites with Pollution-Causing Activities in the EU

In the present work, all sites with pollution-causing activities were collected in a database. The database differentiates two primary categories: the E-PRTR and the national and EU-wide inventories. The latter category consolidates EU-wide databases and national inventory data into a single group (Figure 1). A total of 3664 potentially contaminated sites were identified. Most of these potentially contaminated sites (3291) were derived from national and EU-wide inventories encompassing all pollutants considered in this study. The deposition/application of compost or sewage sludge on fields for PFAS and landfilling and waste handling of PFAS, could not be identified in the datasets and were therefore not included in the mapping. There may be a number of sites with these activities have occurred/still occur in the EU. Among all potentially contaminated sites, the use of fire-fighting foam at airports, military sites, and firefighting training facilities was reported at 2581 sites, representing the highest numbers. This activity is excluded from Figure 1 for clarity, and all other activities are shown.

**Figure 1:**
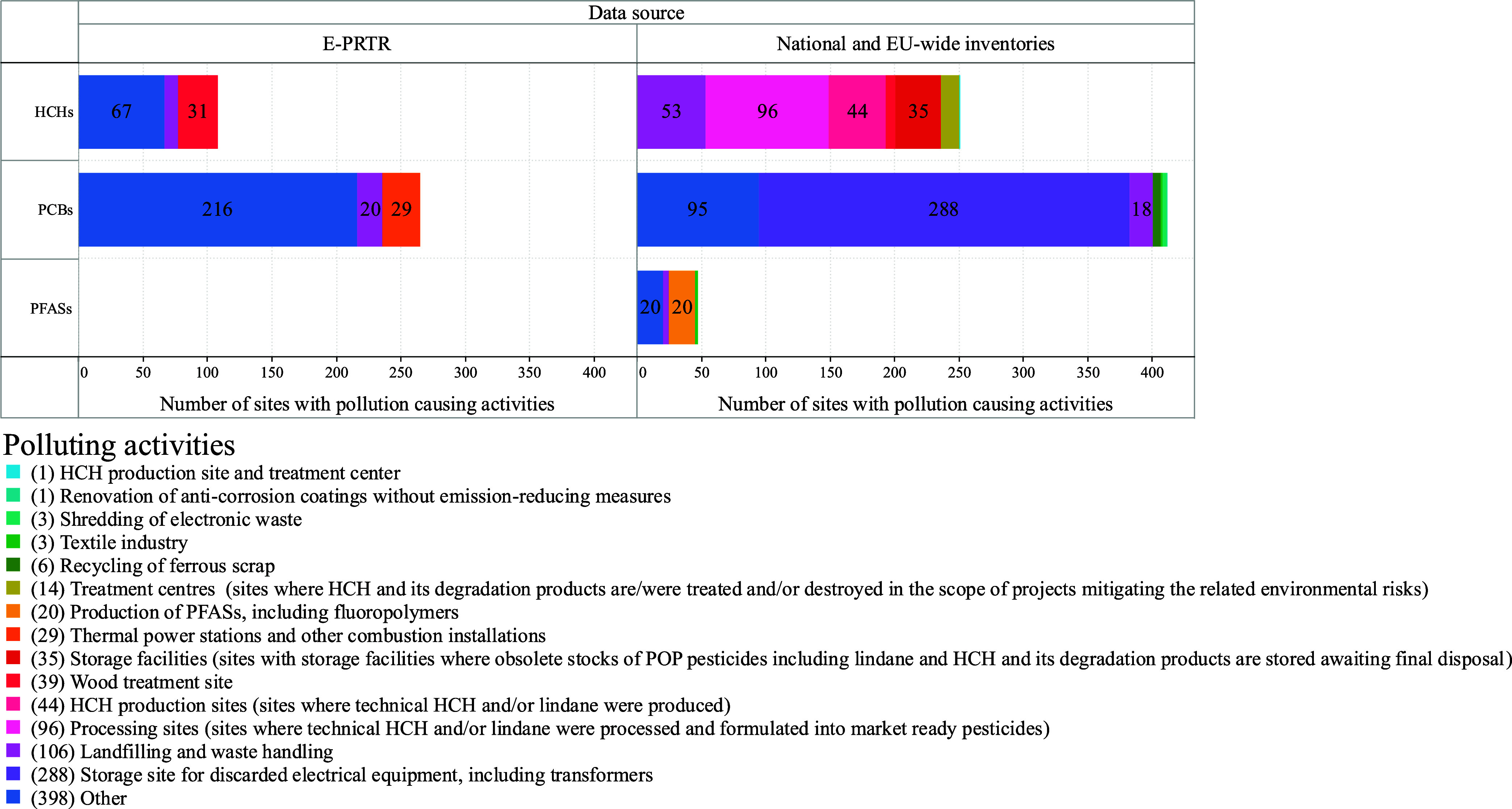
Data sources (E-PRTR and national and EU-wide repositories), and the number of potentially contaminated sites due to the presence of pollution activities from each source for PCBs, HCHs, and PFASs. The numbers in the legend in brackets indicate the number of sites.

Among all of the potentially contaminated sites causing PFAS pollution, Belgium accounted for the highest proportion, with 32% (843 sites) of the total sites, followed by Norway with 12% (310 sites), as shown in Figure 2. These countries have the most data in the Forever Pollution Project database regarding firefighting training and reported incidents. In the case of PCB pollution sources, Luxembourg has the highest number of sites with identified polluting activities, followed by Italy. For HCHs, most of the activities related to HCH contamination, approximately 65% (234), are concentrated in five countries, listed in decreasing order as Germany, Spain, France, Italy, and the Netherlands.

**Figure 2:**
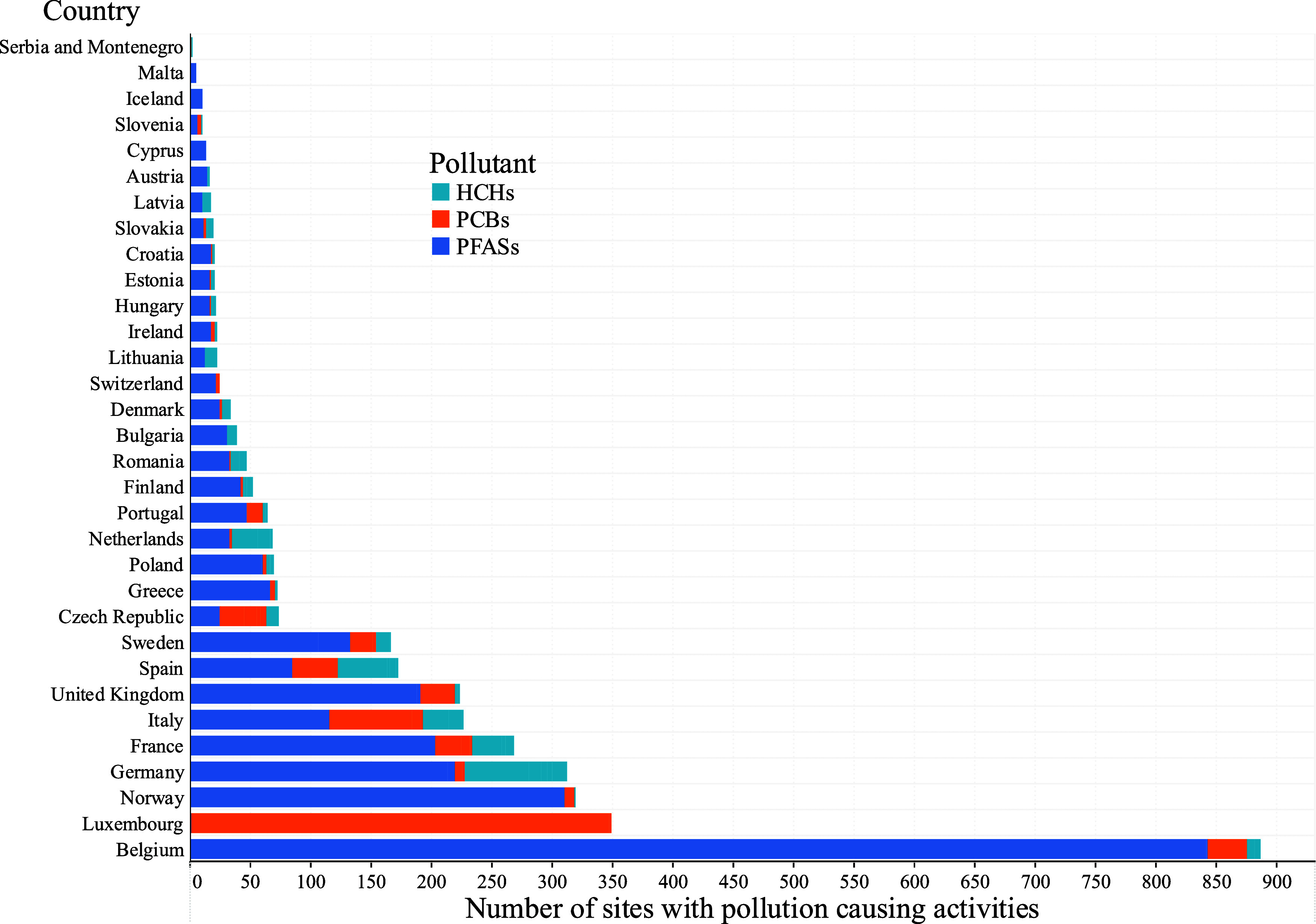
List of the countries and number of sites with identified pollution-causing activities for PCBs, HCHs, and PFASs from all of the data sources included in this study.

#### 3.3 Spatial Distribution, Co-Occurrence of Polluting Activities and Cocontamination

The spatial distribution map provides a visualization of soil-polluting activities across the EU in Figure 3. An online version of this map is also available (https://experience.arcgis.com/experience/b250d33a8c9c4ade9a3ace23ec073ee6). The use of spatial data analysis and mapping to identify clusters of pollution sources may raise concerns regarding potential errors arising from methodological simplifications and data inconsistencies, such as more extensive data from certain countries and less from others. However, validation against historical contamination reports and documented industrial activities associated with PCBs, PFASs, and HCHs is possible and supports the accuracy and reliability of the identified spatial clusters based on available data.

**Figure 3:**
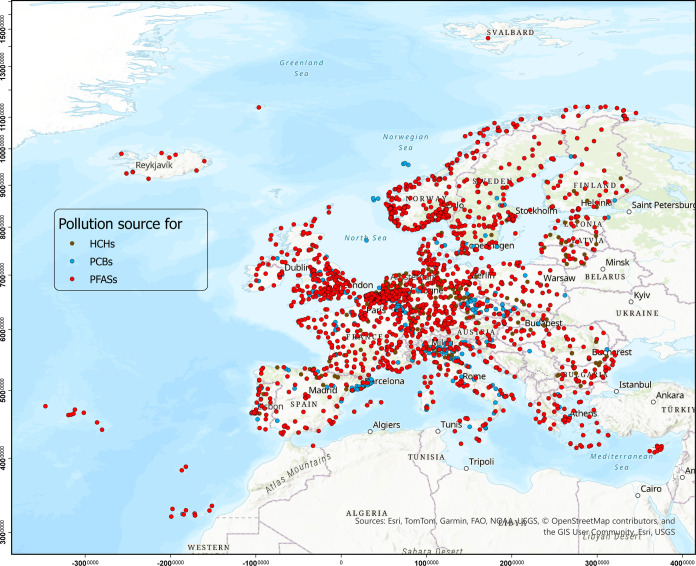
Spatial distribution of the sites with soil pollution-causing activities for PCBs, HCHs, and PFASs.

Most activities identified with PCB contamination are predominantly located in Luxembourg and in the northern region of Italy. These findings align closely with previous studies documenting the spatial distribution of Italian industrial districts.^57^ Maggi et al. indicated that 62.31% of total manufacturing industries in Italy are located in northern Italian regions, predominantly comprising electromechanical industries and textile manufacturing.^57^ Notably, these sectors have been documented in the literature as pollution sources of PCBs and PFASs. Another significant cluster identified in Germany was in the Bitterfeld region, comprising 41 HCHs production and processing sites. Historically, this area was a major industrial hub and experienced extensive contamination due to intensive lindane production from 1951 to 1982, as reported in 2024 in a case study under the Stockholm Convention on Persistent Organic Pollutants (POPs).^58^ Moreover, the region has a longstanding history of broader chemical industry activities dating back to 1893, involving pollutants such as chlorinated solvents, herbicides, and other POPs, which further exacerbated the environmental impact of soil contamination. The former mining pits in this area were also repurposed as waste disposal sites for HCH residues without proper impermeable barriers, leading to prolonged environmental contamination. Previous studies reported significantly higher values of HCHs in the area, which were probably caused by emissions from the former chemical plant Bitterfeld–Wolfen and the landfill.^58,59^

The distribution of PFAS-related activities is diverse; however, there are two clusters in the UK and Belgium, with the PFAS pollution in the latter being more concentrated and visible, particularly around firefighting training centers in Flanders. The validity of this cluster in Flanders, Belgium, for PFAS contamination sources is supported by recent reports. Over 220 PFAS-contaminated sites have been officially documented in this region.^60^ The sources of contamination are the use of PFAS-containing firefighting foam, particularly at firefighter training facilities and major fire incidents. Clear PFAS sources are also evident in Norway, owing to municipal firefighting training centers where aqueous film-forming foam (AFFF) was used in the past and, in some cases, is still in use. Therefore, despite inherent simplifications in the analysis, spatial mapping and clustering effectively capture and highlight significant regional pollution patterns.

It is crucial to acknowledge that the number of sites with pollution-causing activities incorporated into the database from each country varies considerably. This variation is primarily attributable to the differing levels of data availability across the national inventories. However, the ability to visualize spatial patterns supports the identification of sensitive regions that require extensive investigation and remediation, as mentioned in other studies.^61^ By clearly identifying areas with a high likelihood of multiple pollutants based on pollution sources, the present study offers valuable insights into regions that pose elevated environmental and human health risks. Such spatial mapping is critical for prioritizing remediation actions and optimizing the monitoring programs.

Additionally, the co-occurrence of these activities is also clear in an area of 1 km^2^, as shown in Figure 4. Among all of the sites, 2802 source activities occur as singles within a 1 km^2^ area, whereas 333 squares contain two pollution sources, and 52 contain more than two pollution sources within a 1 km^2^ area. The co-occurrence pattern in Figure 4 mirrors the clustering seen in Figure 3, indicating potentially polluted sites in Luxembourg, northern Italy, the UK, Norway, Belgium, and eastern Spain. In eastern Spain, the primary sources of pollution are waste-handling and waste-treatment plants, which result in contamination by HCHs and PCBs.

**Figure 4:**
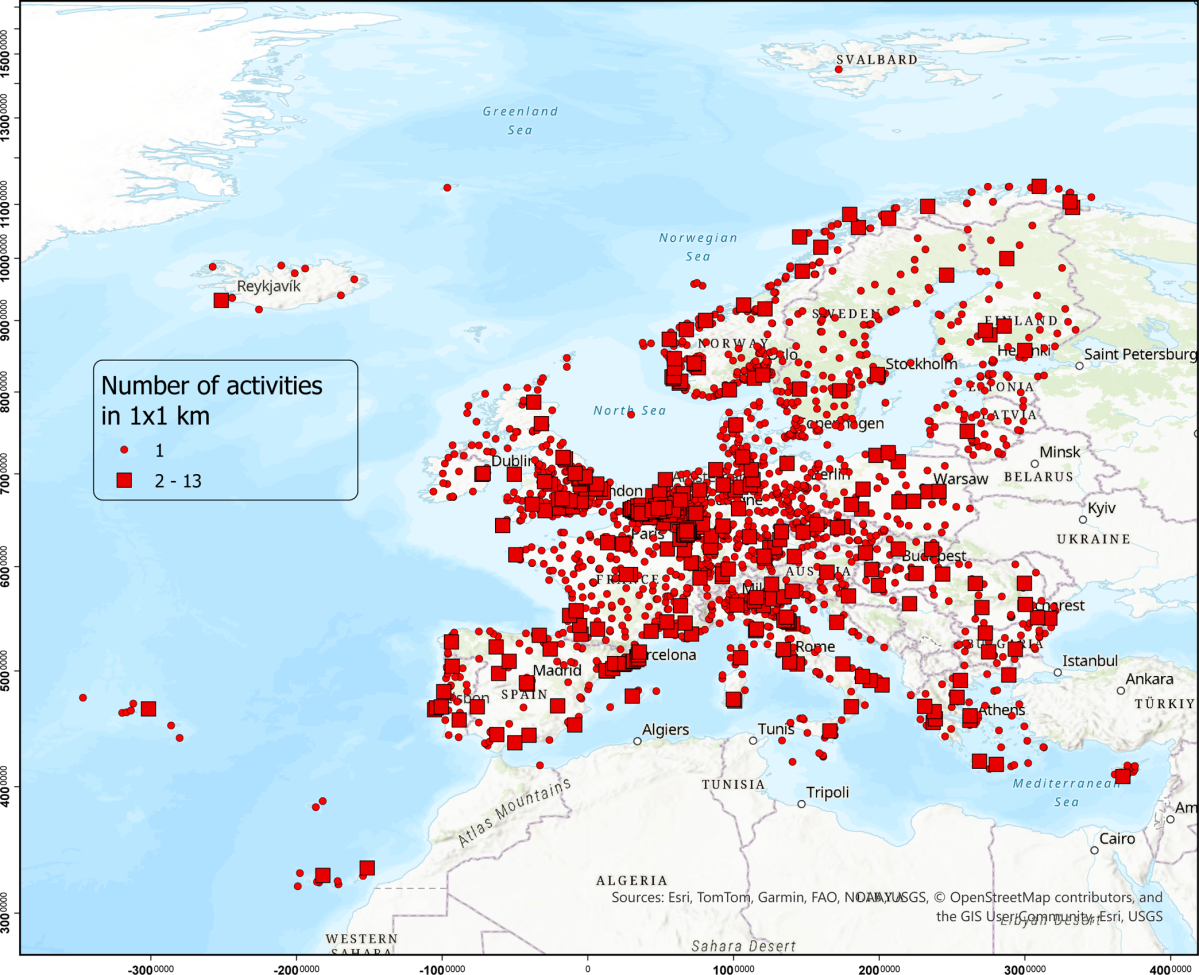
Recorded sites with pollution-causing activities across Europe mapped on a 1 km × 1 km grid. Red dots mark cells with a single activity; red squares mark cells with two or more (up to 13) activities in a 1 km^2^ area.

There are 385 squares on the map where pollutant sources for PCBs, HCHs, or PFASs co-occur within an area of 1 km^2^. However, the co-occurrence of the number of sources alone does not make it clear whether the presence of more than one pollution source contributes to soil contamination by multiple pollutants. Figure S1 further illustrates the details of the presence of co-occurring activities causing pollution of single or multiple pollutants, i.e., cocontamination (meaning the presence of pollution sources related to more than one pollutant).

While many squares show co-occurrence of pollution activities related to a single pollutant, there are also around 10% of the 385 squares where the pollution sources cause pollution of multiple pollutants. The highest number of squares identified were associated with four pollution sources potentially causing cocontamination by PFASs and HCHs; these sites served as production locations for both contaminants. Next, two squares with three sources exhibited cocontamination by PCBs along with HCHs and PCBs with PFASs. However, across 38 squares, the co-occurrence of two polluting activities and the associated potential for soil cocontamination is most observed for PCBs and HCHs. This is followed by PFASs with PCBs at four sites and PFASs with HCHs occurring at a single site.

Recently, research on soil cocontaminants has gained more attention due to advancements in the remediation of sites contaminated with single pollutants, providing a strong base for studying more complex cocontamination of pollutants.^62^ However, mapping pollution-causing sources alone cannot explain the pollutant interactions in soil and is not an element but a prerequisite of risk assessment. Despite these limitations, maps of multiple pollution sources remain crucial as diagnostic tools for identifying and visualizing high-risk areas.^61^ For example, research conducted in Bulgaria used hierarchical cluster analysis (HCA) and principal component analysis (PCA) to classify soil sampling sites. These analyses were used for the creation of shaded maps of spatial patterns of cocontamination from multiple anthropogenic sources, highlighting areas where industrial, agricultural, and vehicular pollutants overlapped within the study area.^63^ Thus, while not sufficient alone, spatial maps form a necessary foundation that must be combined with direct soil analyses, continuous monitoring, and comprehensive evaluations to effectively manage complex environmental-contamination scenarios. Furthermore, the identification of cocontamination hotspots supports the development of integrated remediation strategies that can address multiple pollutant classes simultaneously, potentially reducing overall remediation costs and minimizing soil disturbance compared to sequential treatment approaches.

The spatial co-occurrence and cocontamination mapping in Figures 4 and S1 identifies areas where pollution activities intersect, but it does not explicitly verify actual pollutant concentrations, environmental transport, or bioavailability. Consequently, the spatial clustering of pollution activities identified and the co-occurrence do not provide an estimate of the environmental risks or health effects. On the other hand, these are the currently available data from the E-PRTR, the Forever Pollution Project, and the national inventories^36,37,41^ and represent an important and relevant basis for actual risk assessments. It is highly likely that there are additional locations where polluting activities have taken place; the identification of additional sites will require detailed investigations in terms of the history of sites or further measurements in the soil.

While our spatial analysis effectively identifies areas of potential cocontamination, it necessarily simplifies the complex interactions that occur when multiple pollutant classes are present simultaneously. Research on cocontamination scenarios demonstrates that pollutant interactions in soil can significantly alter individual compound behavior, affecting bioavailability, transport characteristics, and remediation efficacy.^64^ For instance, studies on cadmium–arsenic cocontamination show that the presence of multiple metals can alter cellular partitioning and increase overall plant uptake through synergistic mechanisms. Furthermore, the co-occurrence of the activities causing cocontamination by HCHs, PCBs, and PFASs presents challenges due to the distinct physicochemical properties and environmental fate processes of these chemicals. While PCBs and HCHs are legacy contaminants with relatively well-established degradation pathways, PFASs are essentially nondegradable under natural environmental conditions, which is why they are often called “forever chemicals”. This temporal mismatch means that areas showing historical industrial activity may present ongoing PFAS contamination even after legacy compounds have degraded or been remediated. Therefore, nontargeted screening of soil is essential to identify pollutants in these areas. Studies using nontarget screening approaches have identified numerous previously unknown PFASs in contaminated soils, with traditional target analysis covering less than 23% of the occurring PFASs.^65^ This analytical limitation suggests that our source-based mapping approach can provide a more comprehensive basis for risk assessment than direct soil analysis for PFASs, at least until analytical capabilities and capacities improve. Therefore, despite limitations, the current results effectively serve as a proof of concept, clearly demonstrating that pollution-causing activities can co-occur spatially, posing significant risks of cocontamination. Furthermore, if additional country-specific data from all EU member states become available, the observed clustering and spatial co-occurrence of HCHs, PCBs, and PFASs causing pollution activities will become even more well-documented. Future studies could build on this foundational approach by incorporating additional site-specific pollutant data to better understand and manage cumulative environmental risks.

## Supplementary Material



## Notes

Table S1: The results from national programs that have identified potentially contaminated sites. Figure S1: map and details of the number of sites for co-occurring pollution-causing activities (PDF)

## †

Section Industrial Chemicals, Swiss Federal Office for the Environment, 3011 Bern, Switzerland
