# Supplementary material for: Identification and Spatial Analysis of Co-Occurring Pollution Sources of HCHs, PCBs, and PFASs in the EU and Potential Risks of Soil Pollution
Source: Environ Sci Technol. 2026 May 23;60(22):16158–67. doi: 10.1021/acs.est.6c01518 (PMC13262061; doi:10.1021/acs.est.6c01518)
Supplement: Supplementary file 1 [file es6c01518_si_001.pdf]

Identification and spatial analysis of co-occurring pollution sources of HCHs, PCBs, and PFASs in the EU and potential risks of soil pollution

Naila Hina[1*], Juliane Glüge[1,2], Martin Scheringer[1]
[1]*Institute of Biogeochemistry and Pollutant Dynamics, ETH Zürich, 8092 Zürich, Switzerland*
[2]*present address: Section Industrial Chemicals, Swiss Federal Office for the Environment, 3011 Bern, Switzerland*
*Corresponding Author: *Naila Hina:* nahina@ethz.ch

Table S1: EU and EFTA Member States + UK and whether they have described substance-specific activities that were used to identify potentially contaminated sites. The numbers of potentially contaminated sites were taken from EIONET (2022). These are the numbers that have been registered (not the estimated ones). The estimated numbers are most often higher than the registered ones. If the estimated numbers have been used, this is indicated in brackets.

State	Number of potentially contaminated sites identified?	Are there polluting activities defined that were used to identify the potentially contaminated sites?	Reference
Austria	68,569	A few general polluting activities have been defined; they are not substance specific	1, 2
Belgium (Flanders)	68,000	Polluting activities have been defined, some of them are also substance specific	1, 3
Belgium (Wallonia)	21,064 (estimated)	24 general polluting activities have been defined; they are not substance specific	1, 4
Bulgaria	26	General polluting activities have been defined; they are not substance specific	1, 5
Croatia	2264	There is no official inventory of (potentially) contaminated sites. Some inventories/ databases were developed for specific projects but cannot be considered official since they were not regularly updated and legally set out.	1
Cyprus	84	Substance-specific polluting activities have been identified	1, 6
Czech Republic	29,286	General polluting activities have been defined; they are not substance specific	1
Estonia	300	No information on the pollution activities could be retrieved	1
Finland	26,200	General polluting activities have been defined; they are not substance specific	1, 7
France	6478 (300,000 estimated)	Inventory of emission sources is available for PFASs. No overview information available on the other substance groups and their associated activities. But detailed reports from each	1, 8, 9

		contaminated site (soil and water) with pollution activity and associated substances available	
Hungary	5375	No information on the pollution activities could be retrieved	
Italy	22,274	No list of potentially polluting activities used for the identification of contaminated sites	[1]
Lithuania	12,341	General polluting activities have been defined; they are not substance specific. Substance specific information is available for the monitored sites.	[10]
Luxembourg	12,000	It seems that polluting activities do exist, however we would need to contact the local authorities first, to get access to the system	[11]
Norway	650	Substance-specific polluting activities have been identified	[12]
Poland	none	Substance-specific polluting activities have been identified	[13]
Sweden	83,000	Substance-specific polluting activities have been identified	[14]
Switzerland	38,000	Substance-specific polluting activities have been identified	[1, 15]
The Netherlands	615,000	According to JRC (2018), over a thousand different polluting activities were identified. However, we were not able to find the specific information online	[1, 16]
United Kingdom	21.000 (estimated)	It seems that they were no hard criteria how the sites were identified	[1, 17]

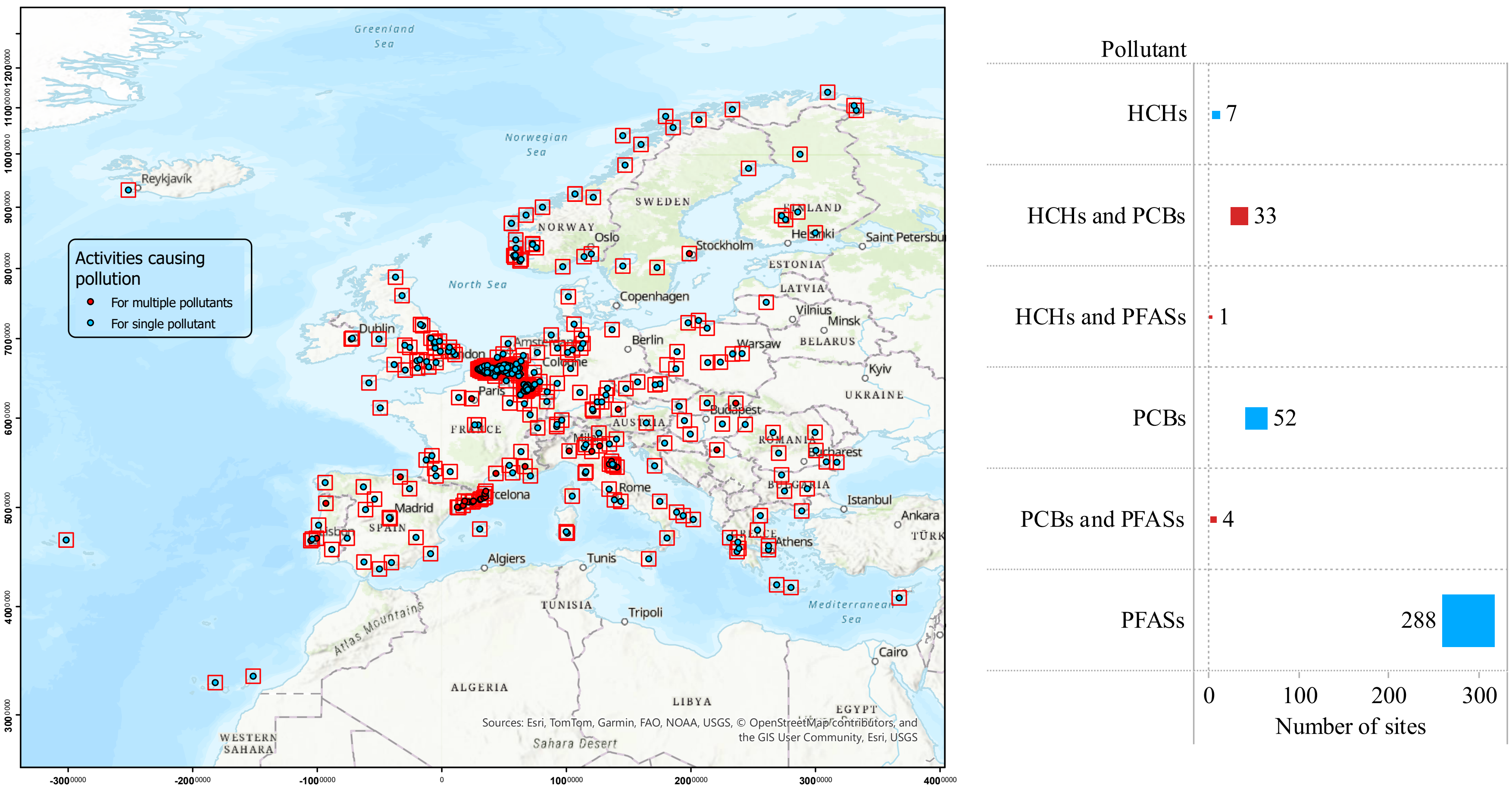

Figure S1 Left: Co-occurred pollution-causing activities mapped on a 1km² grid. Red squares with red dots on the map mark grid cells where co-occurred activities causing pollution of multiple pollutants; blue dots mark cells where all the co-occurred activities in 1km² causing pollution of one pollutant. Right: The blue squares show activities causing pollution by single pollutants, while red squares show activities contributing to multiple pollutants. Square size and the numbers next to the boxes in the plot represents the number of polluting activities co-occur within a 1km² area. The ‘Pollutants’ column lists the names of the pollutants, either as single or in multiple pollutants contamination.

References

(1) JRC (Joint Research Centre). *Status of local soil contamination in Europe.*; 2018. DOI: 10.2760/093804.
(2) European Parliament. Proposal for a Directive of the European Parliament and of the Council establishing a framework for the protection of soil and amending Directive 2004/35/EC. 0086. 2006.
(3) LAREBO - Annex I. List of risk establishments that started operating before 1 June 2015. . https://navigator.emis.vito.be/detail?woId=23569 (accessed Jan, 2025).
(4) Wallonia. Décret relatif à la gestion des sols. 2008.
(5) Bulgaria. Act on Liability for Preventing and remedying ecological damage. 2017.
(6) Demetriades A, A. N., Kaminari M, Vergou K. National inventory of potential sources of soil contamination in Cyprus. Report to Cyprus Ministry of Agriculture, Lefkosia. 2006.
(7) Ministry of the Environment. National risk management strategy for contaminated land in Finland. 2017.
(8) Cavelan A; Togola A. *Inventory of direct sources of PFAS emissions.*; 2024.
(9) Soil pollution, SIS and former industrial sites. https://www.georisques.gouv.fr/risques/basias/donnees/carte#/admin/fxx (accessed 05.05.2025).
(10) Lithuanian Geological Service. https://lgt.lrv.lt/epaslaugos/index.xhtml# (accessed April, 2025).
(11) Luxembourg. Management of polluted sites. 2020.
(12) Miljodirektoratet. Ground contamination - industries and substances (M-813). 2017.
(13) Poland. Regulation on the manner in which the assessment of surface contamination is to be carried out. Item 1395. 2016.
(14) Swedisch EPA. Industry list of contaminated sites (2024). 2024.
(15) BUWAL. *Altlasten Kataster - Erstellung des Katasters der belasteten Standorte.*; 2001.
(16) Rijkswaterstaat. Into Dutch Soils. 2014.
(17) Environment Agency. Dealing with Contaminated land in England: A review of progress from April 2000 to December 2013 with Part 2A of the Environmental Protection Act 1990. 2016.
